# Value of CT and three-dimensional reconstruction revealing specific radiological signs for screening causative high jugular bulb in patients with Meniere’s disease

**DOI:** 10.1186/s12880-020-00504-0

**Published:** 2020-08-31

**Authors:** Junjiao Hu, Anquan Peng, Kai Deng, Chao Huang, Qin Wang, Xueying Pan, Wei Liu, Zhiwen Zhang, Wenqi Jiang, Yichao Chen

**Affiliations:** 1 Department of Radiology, The Second Xiangya Hospital, Central South University, 139 Middle Renmin Road, Changsha, 410011 Hunan China; 2 Department of Otolaryngology-Head and Neck Surgery, The Second Xiangya Hospital, Central South University, Changsha, 410011 Hunan China

**Keywords:** High jugular bulb, Meniere’s disease, Computed tomography three-dimensional reconstruction, Vestibular aqueduct, Endolymphatic hydrops

## Abstract

**Background:**

The aim of the present study was to investigate the pathological features of vestibular aqueduct (VA) related high jugular bulb (HJB) and explore the possible cause-consequence relation between HJB and endolymphatic hydrops (EH), and the potential specific radiological signs for screening causative HJB in Meniere’s disease (MD).

**Methods:**

High-resolution computed tomography (HRCT) and three-dimensional reconstruction (3DRC) were used to detect the anatomical variables associated with VA and jugular bulb (JB) in hydropic and non-hydropic ears. The presence or absence of EH in the inner ear was determined by gadopentetate dimeglumine-enhanced magnetic resonance imaging. The presence of different types of HJB, the anatomical variables of the VA and JB and the three types of anatomical relationship between the VA and HJB were compared between the hydropic and non-hydropic ears using the χ^2^ or Fisher’s exact tests. *P* < 0.05 was considered to indicate a statistically significant difference.

**Results:**

JB was classified as: Type 1, no bulb; type 2, below the inferior margin of the posterior semicircular canal (PSCC); type 3, between the inferior margin of the PSCC and the inferior margin of the internal auditory canal (IAC); type 4, above the inferior margin of the IAC. There were no significant differences in the presence of types 1, 2 and 3 JB between two groups. The presence of type 4 JB, average height of the JB and prevalence of the non-visualization of the VA in CT scans showed significant differences between two groups. The morphological pattern between the JB and VA revealing by 3DRC was classified as: Type I, the JB was not in contact with the VA; type II, the JB was in contact with the VA, but the latter was intact without obstruction; type III, the VA was obliterated by HJB encroachment. There were no significant differences in the presence of type I and II between two groups. Type III was identified in 5 hydropic ears but no non-hydropic ears, with a significant difference observed between the two groups.

**Conclusion:**

The present results showed that JB height and non-visualization of the VA on Pöschl’s plane could render patients susceptible to the development of EH. A jugular bulb reaching above the inferior margin of the IAC (type 4 JB) could obstruct VA, resulting in EH in a few isolated patients with MD. VA obliteration revealed by 3DRC, as a specific radiological sign, may have the potential for screening causative HJB in MD.

## Background

Endolymphatic hydrops (EH) is the pathological substrate of Meniere’s disease (MD), which may be caused by deficient absorption in the sac or obstruction of the endolymphatic duct [1–4]. High jugular bulb (HJB), one of the most common anatomical variant in temporal bone, has been reported to be more common in MD, and it has been suggested to put pressure on the endolymphatic sac (ES) and distal vestibular aqueduct (VA), which contains the endolymphatic duct and the infratemporal endolymphatic sac, resulting in endolymphatic hydrops and Meniere-like symptoms [5, 6]. Although numerous radiological studies have shown smaller VA dimensions and high rate of JB abnormalities among affected individuals, suggesting that HJB is likely to interfere with VA, causing endolymphatic sac dysfunction and the development of EH [7, 8]. However, with the exception of certain case reports, which surgically demonstrated that HJB could obstruct the VA [9, 10], most studies only identified a nonspecific radiological sign, such as JB-related VA dehiscence and JB diverticulum, that did not hold great potential for providing a clinically meaningful understanding of the association between the VA and HJB [11, 12]. The exact role of HJB in the etiology of MD is unclear. Moreover, as the occurrence of HJB was reported at a frequency of 4.6 to 57% in the published literature with the different reference levels above which the jugular bulb was defined as high riding [13, 14], the lack of consensus over the description of HJB and its definition is a problem that has resulted in the existence of different interpretations of HJB. A definitive or consensus classification system for JB is critical to uniformly evaluating causative HJB in the development of EH. The aim of present study was to detect the presence of different types of HJB by high-resolution computed tomography (HRCT), according to the Manjila and Semaan classification of JB [15], identify the anatomic variables of the VA and JB in hydropic and non-hydropic ears [as confirmed by gadopentetate dimeglumine-enhanced magnetic resonance imaging (Gd-MRI), and explore the potential of a specific radiological sign, such as the absence or obstruction of VA caused by HJB, for screening causative HJB underlying the development of EH in MD using CT and three-dimensional reconstruction (3DRC).

## Materials and methods

**Fig. 1:**
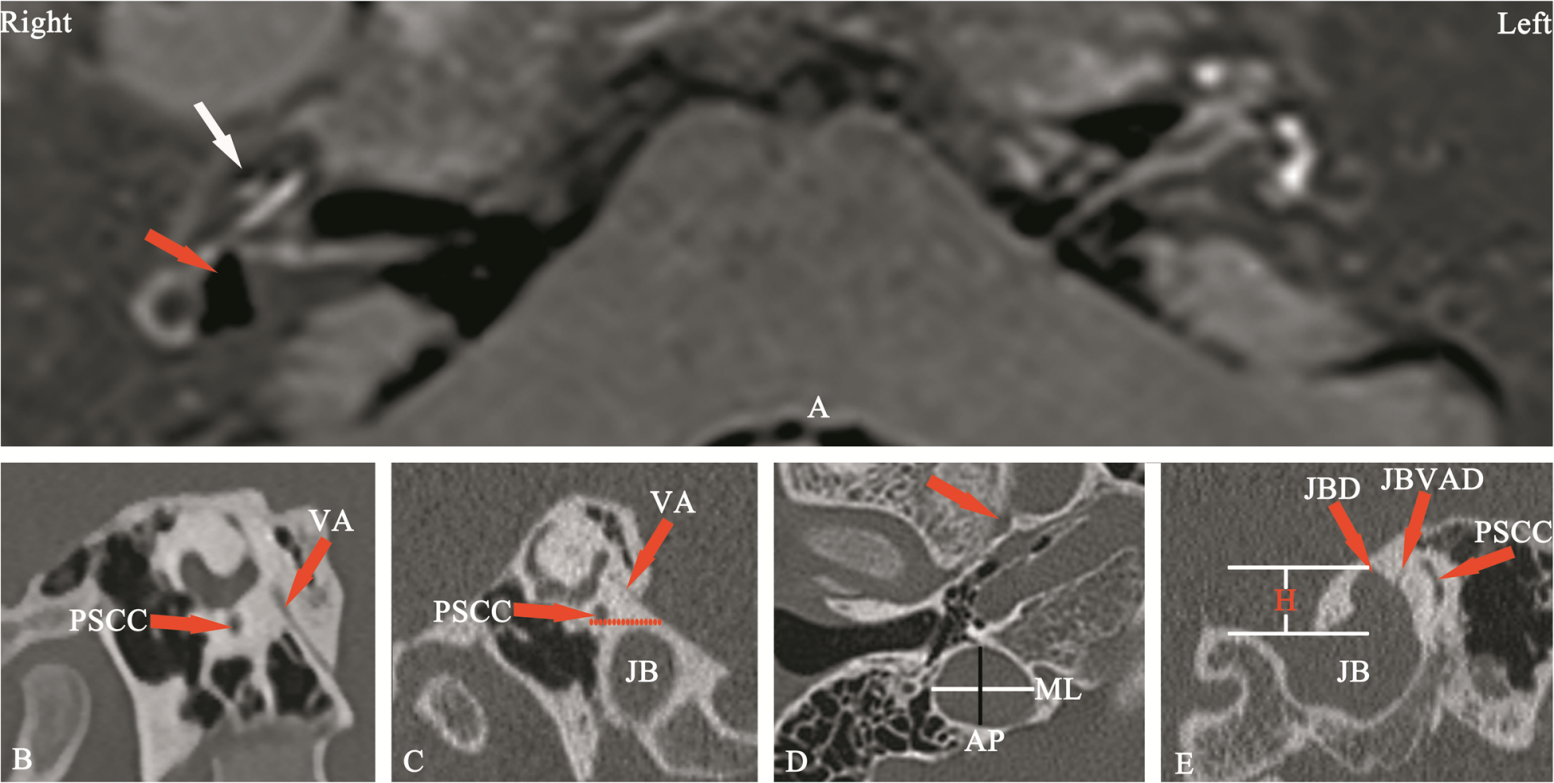
**a** 3D real inversion recovery sequence MRI showing a mild cochlear EH (white arrow) and a significant vestibular EH (red arrow) in the right ear and there is no pathological finding in the left ear. **b** The external aperture of the VA can be well visualized in the Pöschl plane. When failing to visualize this structure, non-visualization of the VA was recorded. In this image, JB was classified as type 1 because of no bulb. **c** The external aperture of the VA can also be well visualized in the Pöschl plane. In this image, JB was classified as type 2 because of the JB below the inferior margin of the PSCC. **d** Anteroposterior (black line) and mediolateral (white line) diameters of the JB were recorded on the axial image at the level where the foramen spinosum (arrow) could be observed. **e** The height of the JB was recorded by measuring the distance between the level of the JB dome and the line passing through the confluence of the sigmoid sinus with the JB on the coronal image. The JBVAD was defined as when the bony coverage separating the JB and VA is dehiscent. The JBD was defined as when a prominent protrusion or outpouching of the JB can be clearly distinguished from a smooth ellipsoidal form on the coronal image. EH, endolymphatic hydrops; JB, jugular bulb; JBD, JB diverticulum; JBVAD, JB-vestibular aqueduct dehiscence; H, height; AP, anteroposterior; ML, mediolateral; PSCC, posterior semicircular canal; VA, vestibular aqueduct

The study was conducted at a large tertiary referral center for vestibular disorders. According to the criteria for Meniere’s disease jointly formulated by the Classification Committee of the Bárány Society [16], from January 2018 and December 2019, ninety-five patients with the clinical diagnosis of intractable definite and probable MD were referred for 3 T MR imaging of the temporal bone to demonstrate EH and to exclude other causes of vertigo and hearing loss such as vestibular schwannoma using intravenous and intratympanic administration of gadopentetate dimeglumine (IV-Gd + IT-Gd) (Fig. 1a). All patients referred for MRI were evaluated by two experienced otologist (AQ. Peng and Q. Wang) and performed CT scan during the same time period. Patients aged < 18 years (*n* = 2) or with a history of inflammatory otitis media (*n* = 1), temporal bone neoplasm or trauma (*n* = 0), congenital ear anomalies (*n* = 0), or previous otologic surgery (*n* = 1) were excluded from the study. A total of 91 patients met the inclusion criteria. All images obtained by HRCT and MRI were evaluated by two experienced radiologists who were blinded to the diagnosis of all patients. If their evaluations differed, a third radiologist made the final decision. Anatomical aspects of the JB and the positioning and structure of the VAs were systematically analyzed.

The present study was approved by the Medical Ethics Committee of the Second Xiangya Hospital (certificate number: S452), and all patients who participated provided their written informed consent. According to the Manjila and Semaan classification of JB location [15], JB was classified as: Type 1, no bulb (Fig. 1b); type 2, below the inferior margin of the posterior semicircular canal (PSCC) (Fig. 1c); type 3, between the inferior margin of the PSCC and the inferior margin of the internal auditory canal (IAC) (Fig. 3); type 4, above the inferior margin of the IAC (Fig. 4). The presence of different types of HB was compared between the hydropic and non-hydropic ears. In temporal bone HRCT, the external aperture of the VA could be well visualized on Pöschl’s plane (Fig. 1b and c); when failing to visualize the external aperture of the VA, non-visualization of the VA was recorded. The anteroposterior and mediolateral diameters of the JB were recorded on the axial image at the level where the foramen spinosum could be observed (Fig. 1d). The height of the JB was recorded by measuring the distance between the level of JB dome and the line passing through the confluence of the sigmoid sinus with the JB on the coronal image (Fig. 1e). The JB-related VA dehiscence (JBVAD) was defined as when the bony coverage separating the JB and VA is dehiscent (Fig. 1e). The JB diverticulum was defined as a prominent protrusion or outpouching of JB could be clearly distinguished from a smooth ellipsoidal form on the coronal image (Fig. 1e). The anatomical variables of the VA and JB were compared between the hydropic and non-hydropic ears with HJB.

### Gd-MRI

MRI was performed using a single-dose (0.2 ml/kg) intravenous administration of gadopentetate dimeglumine (Magnevist; Bayer AG) 4 h prior to the MRI scan and intratympanic administration of 8-fold-diluted Gd in both ears 24 h prior to the MRI scan. All scans were performed on a 3 T MRI scanner (Magnetom Verio; Siemens AG) using a 12-channel head coil. 3D real inversion recovery (3D-real IR) sequence MRI images was collected as previously described [17]. Briefly, the parameters for the 3D-real IR sequence were: Voxel size, 0.4 × 0.4 × 0.8 mm; scan time, 14 min; repetition time (TR), 9000 msec, echo time (TE), 181 msec; inversion time (TI), 1730 msec; slice thickness, 0.80 mm; field of view (FOV), 160 × 160 mm; matrix size, 3300 × 918. The off-label use of IT-Gd-MRI was performed after receiving the informed consent. The degree of EH in the cochlea was assessed by visual comparison of the relative areas of the non-enhanced endolymphatic space versus the contrast-enhanced perilymph space in the axial plane. According to the criteria previously described by Nakashima et al. and Wesseler et al. [18, 19], in case of the cochlea, the evaluation was carried out on the mid-mediolar level in regards to a possible dislocation of the Reissner’s membrane. The degree of cochlear hydrops was categorized as none (normal finding without EH), grade I (mild EH), or grade II (significant EH). An EH of the vestibule was determinate by the volume-ratio of endolymphatic space to the total vestibule (endolymph to vestibule-volume ratio) using the syngo.via software package (VB20A, Siemens Healthcare, Erlangen, Germany), none when less than 30%, grade I when 30 to 50%, grade II when more than 50% of the vestibular space was filled with endolymph. According to that described by Wesseler et al [19], this ratio was not estimated based on one section plane alone, but was measured separately in every plane showing the vestibule, and then using the average of those values calculated as the overall result.

### CT scans and 3D reconstruction

**Fig. 2:**
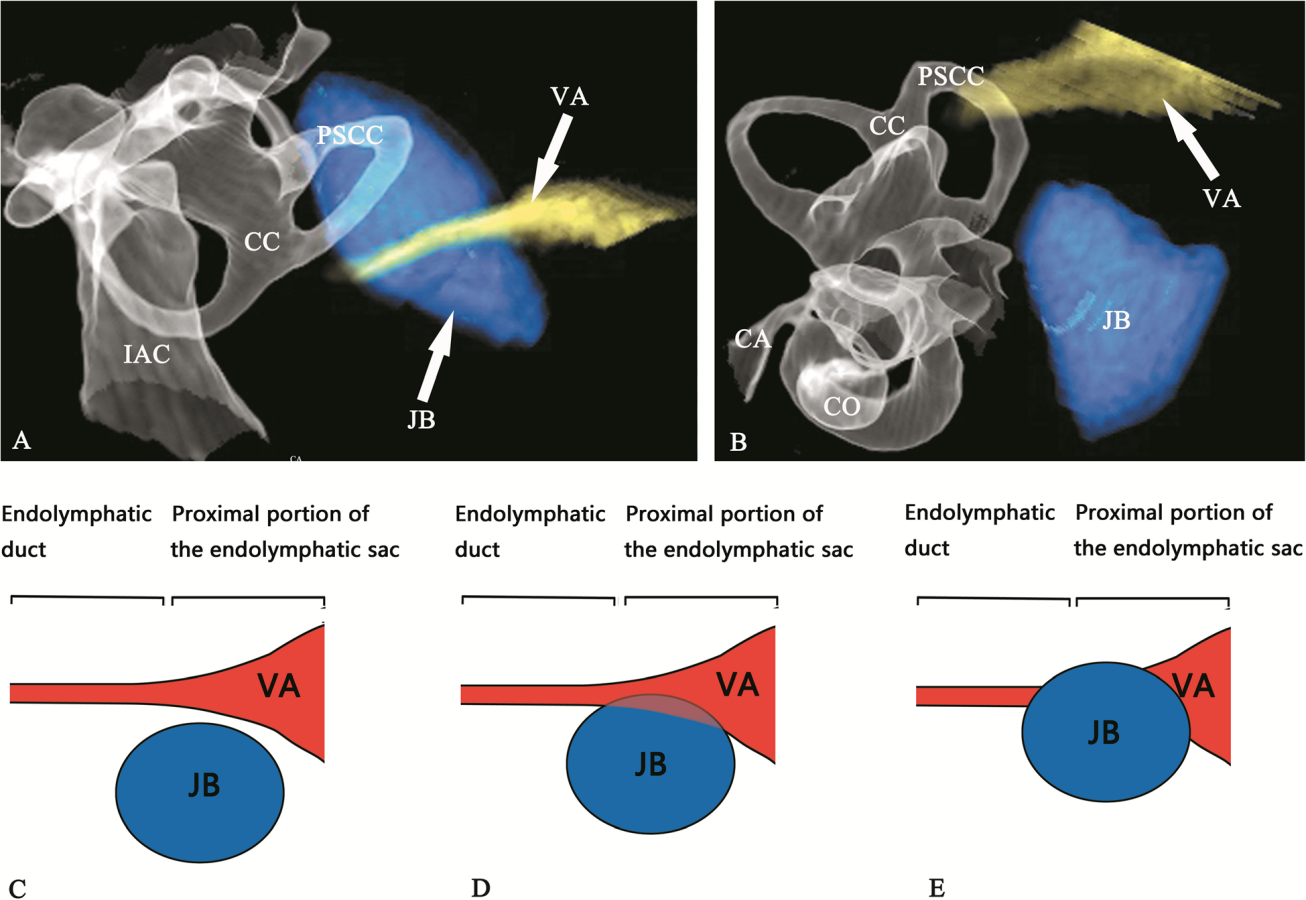
**a** Posterosuperior and **b** posteroinferior view of 3D stereoscopic images showing the normal structure of the inner ear and JB. The pseudocolor technique was used to display the course of the VA between the isthmus and the operculum (yellow) and JB (blue). Drawing showing 3 types of morphological relationships between the JB and VA: Type I, JB is not in contact with the VA, no matter how close or far apart the VA is (**c**); type II, JB is in contact with the VA, but the VA is intact without absence or obstruction (**d**); type III, the VA is obliterated by high jugular bulb encroachment (**e**). CO, cochlea; CA, cochlear aqueduct; CC, common crus; IAC, internal auditory canal; JB, jugular bulb; PSCC, posterior semicircular canal; VA, vestibular aqueduct

HRCT scans were performed using a Somaton Plus 4A CT scanner (Siemens AG) in 91 patients with a clinical diagnosis of MD. The patients underwent cross-sectional imaging with the mandible closed, so that the basal scanning line was parallel to the orbitomeatal line. The images were performed in the helical mode covering the area from the external auditory meatus to the petrous bone. The scanning parameters were as follows: 120 kVs, 100 mAs, 0.75 mm collimation, 1 mm reconstruction increment, a pitch factor of 1 and a field of view of 100 mm. The coronal and axial images of the ears of interest were reconstructed with a 0.1 mm reconstruction increment and a field of view of 50 mm in every case. The topographic relation between the JB and the VA was assessed. The CT scan with 3D reconstruction was performed using the volume rendering technique on the workstation (syngo.via VB10B; Siemens AG). All reformatted images were obtained by a neuroradiology fellow or a postprocessing technologist. The application of different soft-tissue and bone algorithms to the 3D reformation permitted multiprojectional display of the various temporal bone structures. With the use of a built-in 3D cut-plane software technique, individual temporal bone structures were “removed” and analyzed, allowing the optimal display of microanatomic components such as the cochlea, vestibule, semicircular canals, IAC, cochlear aqueduct, VA, and JB. The pseudocolor technique was used to display the VA and JB (Fig. 2a and b).

The anatomical relationship between VA and HJB yielded by the three-dimensional reconstruction (3DRC) was classified into three types, based on their morphological pattern: Type I, JB was not in contact with the VA, no matter how close or far apart the VA was (Fig. 2c); type II, JB was in contact with the VA, but the VA was intact without absence or obstruction (Fig. 2d); type III, the VA was obliterated by HJB encroachment (Fig. 2e). The frequency of the three types of anatomical relationship between the VA and HJB was compared between the hydropic and non-hydropic ears.

For statistical analysis, the χ^2^ or Fisher’s exact tests were used for two-group comparisons. *P* < 0.05 was considered to indicate a statistically significant difference. All data were analyzed with SPSS version 26.0 (IBM Corp.).

## Results

According to Gd-MRI evaluation, a total of 92 ears from 78 patients were diagnosed with endolymphatic hydrops, there were 8 definite bilateral MD (hydropic ears, *n* = 16), 6 definite unilateral MD (hydropic ears both in affected sides and contralateral asymptomatic sides, *n* = 12), 62 definite and 2 probable unilateral MD (hydropic ears in affected sides, *n* = 64). A total of 90 ears from 77 patients were diagnosed with no endolymphatic hydrops, there were 62 definite and 2 probable unilateral MD (non-hydropic ears in contralateral unaffected sides, *n* = 64), 1 patient with definite MD and 12 patients with probable MD (non-hydropic ears in bilateral sides, *n* = 26). High jugular bulb (HJB) was evaluated in the following 2 groups: i) Hydropic ears, including 92 ears from 78 patients (43 females and 35 males; aged 18–78 years; mean age, 48.5 years); ii) non-hydropic ears, including 90 ears from 77 patients (41 females and 36 males; aged 26–78 years; mean age 49.6 years). No statistical difference in age or sex was identified between the hydropic and non-hydropic ears (*P* > 0.05). Table 1 shows the presence of different types of HB based on the Manjila and Semaan classification system for JB [17]. The overall incidence of HJB, including types 2, 3 and 4 was 36 (39.1%) in hydropic and 31 (34.4%) in non-hydropic ears, which was not statistically significant (*P* = 0.512). In addition, no statistically significant differences in the frequency of types 1, 2 and 3 JB were observed between the hydropic and non-hydropic ears (P = 0.512, *P* = 0.556 and *P* = 0.805, respectively). However, a significant difference in the presence of type 4 JB was observed between hydropic and non-hydropic ears (*P* = 0.018). Table 2 shows the anatomical variables investigated in 36 hydropic and 31 non-hydropic ears with HJB, including types 2, 3 and 4. The mean ± standard deviation of the JB height was 9.2 ± 3.4 mm in hydropic and 6.6 ± 2.1 mm in non-hydropic ears. There was a significant difference in the average height of the JB between the values in two groups (*P* = 0.000). The mean ± standard deviation values of the anteroposterior and mediolateral diameters of the JB were 8.2 ± 2.2 mm and 9.2 ± 2.4 mm in hydropic ears, and 8.5 ± 1.9 mm and 8.8 ± 2.2 mm in non-hydropic ears, respectively. There were no differences in the mean anteroposterior (*P* = 0.556) or mediolateral diameters of the JB (*P* = 0.482) between two groups. The non-visualization of the external aperture of the VA in the Pöschl plane was found in 9 hydropic and 2 non-hydropic ears, which was statistically significant (*P* = 0.041). The presence of JB diverticulum or dehiscence with a VA was detected in 11 hydropic and 8 non-hydropic ears. There was no difference in the frequency of JB diverticulum or dehiscence between the two groups (*P* = 0.666). The anatomical relationship between VA and HJB, as determined by 3DRC, was classified into types I, II and III. Type I was identified in 25 hydropic ears and 27 non-hydropic ears, and type II was identified in 6 hydropic ears and 4 non-hydropic ears. There was no statistically significant difference in the presence of types I (*P* = 0,084) and II (*P* = 0.666) between the two groups. Type III was identified in 5 hydropic and no non-hydropic ears. A statistically significant difference was observed in the presence of type III between the two groups (*P* = 0.031). Moreover, all type IIIs were found in hydropic ears with type 4 JB.

**Table 1:** The presence of different types of JB classified by Manjila and Semaan [13] in 92 hydropic ears and 90 non-hydropic ears

	Hydropic ears *n* = 92	Non-hndropic ears *n* = 90	*P*-value
Type 1	56 (60.9%)	59 (65.6%)	P = 0.512
Type 2	21 (22.8%)	24 (26.7%)	P = 0.556
Type 3	7 (7.6%)	6 (6.7%)	P = 0.805
Type 4	8 (8.7%)	1 (1.1%)	P = 0.018

*n* number

**Table 2:** Comparison of variables investigated in 36 hydropic ears and 31 non-hydropic ears showing HJB including type 2, type 3 and type 4

Categorical Variables	Hydropic ears *n* = 36	Non-hydropic ears *n* = 31	*P*-value
Height of the JB (mm), Mean ± SD	9.2 ± 3.4	6.6 ± 2.1	*P* = 0.000
Anteroposterior diameter of the JB (mm), Mean ± SD	8.2 ± 2.2	8.5 ± 1.9	*P* = 0.556
Mediolateral diameter of the JB (mm), Mean ± SD	9.2 ± 2.4	8.8 ± 2.2	*P* = 0.482
Nonvisualization of the Pöschl plane external aperture of VA (n, %)	9 (25%)	2 (6.5%)	*P* = 0.041
Presence of diverticulum or dehiscence with vestibular aqueduct (n, %)	11 (30.6%)	8(25.8%%)	*P* = 0.667
Anatomic relationship between VA and HJB on 3DCT (n,%)			
Type I	25 (69.4%)	27 (87.1%)	*P* = 0.084
Type II	6 (16.7%)	4 (12.9%)	*P* = 0.666
Type III	5(13.9%)	0 (0%)	*P* = 0.031

*n* number, *HJB* high jugular bulb; *JB* jugular bulb

## Discussion

**Fig. 3:**
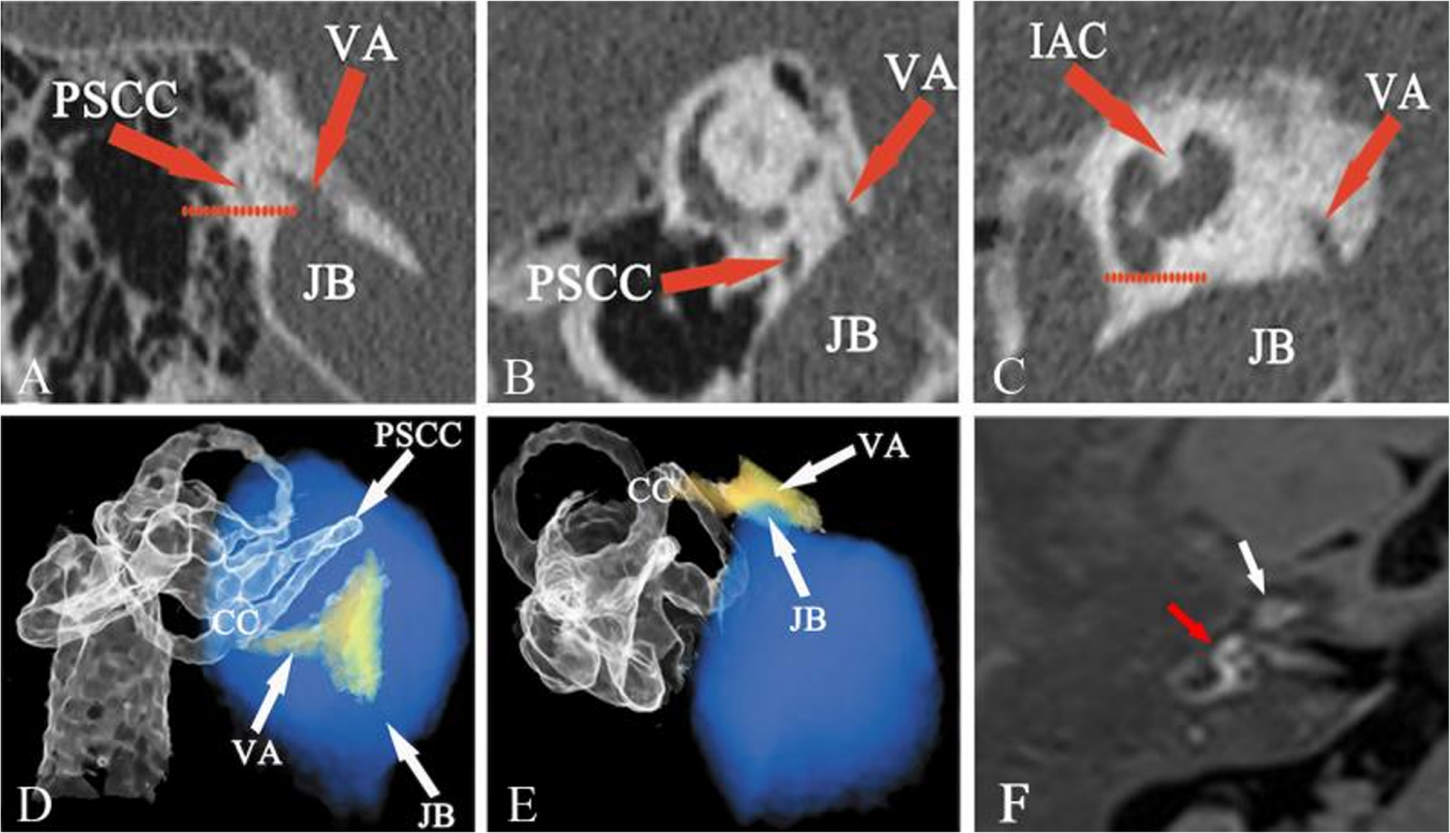
Type 3 JB in a non-hydropic ear. Type 3 is demonstrated in the (**a**) coronal, **b** Pöschl and **c** sagittal planes with the JB above the inferior margin of the PSCC, but below the inferior margin of the IAC, which was imaged at the fundus with the modiolus. A dehiscence was identified between the visualized external aperture of the VA and the upward extension of the JB (**a**, **b** and **c**). Three-dimensional reconstruction computed tomography showing the type II anatomic relationship between JB and VA, where the JB was found to be in close contact with the VA, but the VA was shown intact without obstruction in the (**d**) posterosuperior and (**e**) posteroinferior view. **f** 3D-real IR MRI showing no endolymphatic hydrops in either the cochlea (white arrow) or the vestibule (red arrow), where normal bright perilymphatic fluid was visible. CC, common crus; JB, jugular bulb; JBVAD, JB-vestibular aqueduct dehiscence; PSCC, posterior semicircular canal; IAC, internal auditory canal; VA, vestibular aqueduct; 3D-real IR, three-dimensional real inversion recovery

**Fig. 4:**
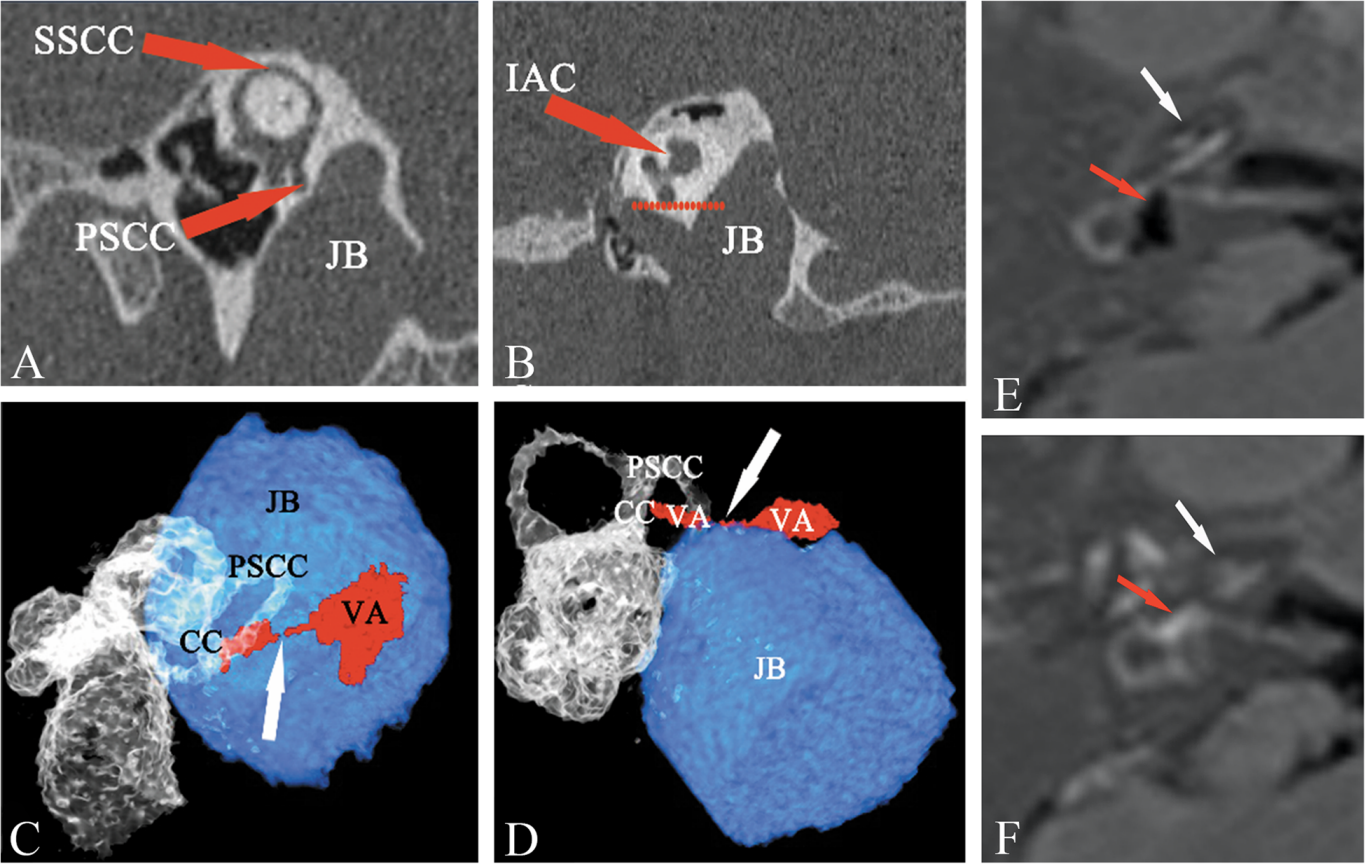
Type 4 JB in a hydropic ear. Type 4 was demonstrated in the (**a**) Pöschl and (**b**) sagittal planes with the JB well above the inferior margin of the PSCC, as well as the inferior margin of the IAC, which was imaged at the fundus with the modiolus. The external aperture of the VA was not visualized in the Pöschl plane (**a**) and sagittal plane (**b**), where an upward extension of the bulb invaded the region of the VA. **c** and **d** Three-dimensional reconstruction computed tomography showing the type III anatomic relationship between JB and VA, where VA obliteration (white arrow) was observed on the posterosuperior view (**c**) and an upward JB encroachment upon the VA resulting in VA obstruction (white arrow) was observed on the posteroinferior view (**d**). 3D-real IR MRI showed an extremely large EH in the vestibule (red arrow) with significant cochlear hydrops (white arrow) before surgery (**e**). Postoperative image showed the cochlear and vestibular hydrops disappeared, where normal bright perilymphatic fluid was visible in both cochlea (white arrow) and vestibule (red arrow) (**f**). CC, common crus; JB, jugular bulb; IAC, internal auditory canal; PSCC, posterior semicircular canal; VA, vestibular aqueduct; SSCC, superior semicircular canal; 3D-real IR, three-dimensional real inversion recovery

To the best of our knowledge, the present study is the first to report the presence of different types of JB, according to the classification by Manjila and Semaan [15], and the anatomical variables detected using HRCT and 3DRC in the ears with the confirmation of endolymphatic hydrops and no endolymphatic hydrops by Gd-MRI, suggesting a potential relationship between the height of JBs and the non-visualization of the VA and the development of EH. There was a likelihood of presence of EH related to HJB in type 4 JB, but not in types 2 and 3. An obliteration of the VA, as determined by 3DRC, could be deemed a specific radiological sign for screening causative HJB in MD. Meniere disease or endolymphatic hydrops has been associated with HJB in several studies [6–8]. As there is no consensus on the exact definition of HJB, the results regarding its role in the etiology of MD are controversial. Redfern et al *and* Park et al have described a higher prevalence of JB abnormalities in patients with MD, as compared with the general population [13, 20]. However, numerous studies have reported no difference in the prevalence of HJB between affected and unaffected ears [6, 21]. In the present study, the Manjila and Semaan classification was used to divide JBs into types1 (Fig. 1b), 2 (Fig. 1c), 3 (Fig. 3a, b and c) and 4 (Fig. 4a and b). A significantly higher prevalence of type 4 JB was found in hydropic ears, as compared with non-hydropic ears, but there were no differences in the presence of type 2 and type 3 JB and overall incidence of HJB between hydropic and non-hydropic ears. It was also identified that the height of JB, and not its size, contributed to the likelihood of the HJB-related endolymphatic hydrops. Therefore, type 4 JB with an upward extension of the bulb to a sufficient height has shown a great potential for the development of EH in some MD patients. As type 4 JB accounted for 8.7% of all JB cases in hydropic ears, the majority of JB cases in hydropic ears didn’t show a cause-consequence relation between HJB and EH.

In addition, the JB abnormalities, such as JB diverticulum (JBD) and JBVAD have been linked to a variety of cochleovestibular symptoms, depending on their impact on surrounding structures [10, 22]. Those without an HJB were more likely to have dehiscence or diverticulum [8]. In the present study, no difference in the frequency of JB diverticulum or dehiscence was observed between hydropic and non-hydropic ears, suggesting there is no association between the presence of JBD and JBVAD, and the development of endolymphatic hydrops.

In addition, as the link between HJB and MD is based on the hypothesis that HJB disturbs the VA to the point of producing hydrops [8, 11], research has focused on how the VA is affected by JB abnormalities. Several studies have radiologically demonstrated hypoplastic VAs and narrowed or obliterated endolymphatic ducts in MD [23, 24]. However, although the majority of the literature has shown a narrow VA, the results of certain studies could be interpreted in different ways. According to Ikeda and Sando, 21% of the normal population has a hypoplastic VA [25]. Sando and Ikeda [26] also reported that 40% of patients with MD have a normoblastic or hyperplastic VA. Therefore, there are conflicting points of view regarding the role of the VA in MD. Considering the complex VA anatomy, the inconsistent results could be attributed to different imaging criteria used for the measurement of the VA. Prior studies have shown that a Pöschl plane image could reveal nearly the entire external aperture of the VA with maximum accuracy, as compared with the conventional axial plane [27]. In addition, as the VA is located between the common crus and PSCC, the current proposal of the Manjila and Semaan classification for JB accounts for the relationship of PSCC; therefore, it is reasonable to postulate that an upward extension of JB above the inferior margin of the PSCC is likely to erode the region of the VA, which could be shown as VA absence (non-visible type) in a Pöschl plane image (Fig. 4a). The present results demonstrated a significantly higher prevalence of non-visualization of the VA in hydropic ears, as compared with that in non-hydropic ears, suggesting that the presence of hydrops may be linked to HJB-caused VA obliteration.

However, non-visualization of the VA was not bound to VA obliteration, a non-visible type aqueduct can occur in normal ears [25, 26]. As VA dimensions and JB abnormalities can vary, only a few of the structures of the VA and HJB could be viewed, and their spatial relationships could not be accurately observed on conventional CT imaging. 3DRC imaging has been used to evaluate VA and membrane labyrinth in MD patients [28, 29] and has yielded more precise images than those generated by conventional CT. In the present study, 3DRC imaging systematically displayed the detailed structures of the temporal bone which enabled us to illustrate the distinct spatial relationship of VA and JB. There was a significant difference in type III relationship of VA and JB between hydropic and non-hydropic ears, suggesting a significant association between VA obstruction and the presence of EH. No significant differences were observed in the prevalence of types I and II between hydropic and non-hydropic ears, suggesting there was no relationship between the JB neighboring VA or JB touching VA and the development of EH. In addition, in type III, 3DRC displayed the upward JB penetration of the VA or encroachment upon the VA, which caused the obliteration of its distal part (Fig. 4c and d) with the presence of EH (Fig. 4e). By contrast, in types I and II, stereoscopic images showed the VA intact by means of varying the angles of view on the computer display (Fig. 3d and e) without the presence of EH (Fig. 3f); this provided insights into the pathogenetic mechanisms underlying the HJB-related development of EH.

As All type IIIs were found in hydropic ears with type 4 JB in our cohort, It can be postulated that the disruption of VA may occur only when the HJB reaches to a sufficient height, such as above the inferior margin of the IAC, and such pathological feature of VA related HJB was likely to be screened with the specific radiological sign revealing by 3DRC. Furthermore, the non-visualization of the VA shown on Pöschl plane occurred in 9 (25%) hydropic and 2 (6.5%) non-hydropic ears, whereas the obliteration of the VA on 3DRC was found in 5 (13.9%) hydropic and no non-hydropic ears. This finding showed a higher accuracy and optimal specificity for evaluating VA obliteration using stereoscopic images.

Additionally, Fig. 4 showed the results of CT and 3DRC, and the dynamic change of EH with Gd-MRI prior to and following surgery in one of five MD patients with visualization of the obstruction of VA on 3DRC images, who was performed endolymphatic sac surgery on the right affected ear. Not surprisingly, the proximal portion of endolymphatic sac was found absent as the encroachment of HJB. Then, the blockage of endolymphatic duct was opened to drain excess endolymphatic fluids. Two weeks following surgery, Gd-MRI showed a complete reversal of hydrops in both cochlea and vestibule (Fig. 4e and f), suggesting that drainage of endolymphatic duct could be an effective means of relieving hydrops. This result further confirmed an obstruction of the VA by HJB was indeed the cause of endolymphatic hydrops in some MD patients. Although the prevalence is low, the identification of this causative factor is very important for both the diagnosis of MD and surgical planning in MD treatment. As discontinuity of the VA, the ES shunting/decompression procedures, which targeted the ES to improve the fluid resorptive functions of the ES, most likely couldn’t work in this patient due to the obliterated VA that separates the ES from the other labyrinthine fluid spaces.

The present study had several limitations. First, the disruption of VA was confirmed surgically in only one patient; in other 4 patients, the lack of surgical or pathological confirmation of VA obliteration shown in Pöschl plane or 3DRC images decreasing the accuracy of the results. Secondly, the radiological finding of an intact VA on 3DRC reflected the results of the morphological evaluation of the VA; whether existence of dysfunction in these intact VA is unknown. A hypothesis has been proposed that the presence of any JB abnormality may contribute to the development of Ménière’s symptoms, presumably by interfering with neighboring inner ear structures to alter the direction of endolymphatic flow or ES venous drainage [8].

Eventually, the presence of JB type 3 and HJB type 4 were scarce among our cohort and larger studies are desirable for further confirmation of current hypothesis.

## Conclusion

In conclusion, the height of JB and the non-visualization of the VA were found likely to be linked to the presence of hydrops. Based on the Manjila and Semaan classification of JB, the frequency of types 1, 2 and 3 JB and the overall incidence of HJB did not show an association with EH. However, type 4 JB reaching above the inferior margin of the IAC could obstruct VA resulting in EH in a few isolated patients with MD. VA obliteration revealed by 3DRC, as a specific radiological sign, may have the potential for screening causative HJB in MD which may be beneficial for surgical planning.

## Data Availability

All the data generated or analyzed during this study are included in this published article or are available from the corresponding author on reasonable request.

## Abbreviations

VA: Vestibular aqueduct
HJB: High jugular bulb
HRCT: High-resolution computed tomography
3DRC: Three-dimensional reconstruction
JB: Jugular bulb
PSCC: Posterior semicircular canal
IAC: Internal auditory canal
EH: Endolymphatic hydrops
MD: Meniere’s disease
ES: Endolymphatic sac
Gd-MRI: Gadopentetate dimeglumine-enhanced magnetic resonance imaging
IV-Gd + IT-Gd: Intravenous and intratympanic administration of gadopentetate dimeglumine
JBVAD: JB-related VA dehiscence
3D-real IR: 3D real inversion recovery
JBD: JB diverticulum
